# Tegoprazan dual and quadruple therapy for *Helicobacter pylori* eradication: a prospective, randomized controlled trial in Beijing, China

**DOI:** 10.3389/fmed.2025.1629567

**Published:** 2025-08-12

**Authors:** Jianping Cheng, Xiaolin Zhao, Chanjuan Fan, Kun Huang, Yong Cai, Zhen Li, Dongling Xie, Lili Zhai, Xiaomei Zhang, Haiou Ding

**Affiliations:** ^1^Department of Gastroenterology, Civil Aviation General Hospital, Beijing, China; ^2^Department of Gastroenterology and Hepatology, The First Medical Center, Chinese PLA General Hospital, Beijing, China; ^3^Department of Pharmacy, Civil Aviation General Hospital, Beijing, China

**Keywords:** *Helicobacter pylori*, tegoprazan, bismuth, dual therapy, quadruple therapy, eradication rate

## Abstract

**Objective:**

The identification of novel and effective treatments for *Helicobacter pylori* (*H. pylori*) infection remains a critical need. Treatment is indicated for peptic ulcer disease, gastric MALT lymphoma, and gastric cancer prevention, following diagnosis via non-invasive testing or endoscopy. This study aimed to investigate the efficacy and safety of tegoprazan-based regimens compared to bismuth-containing quadruple therapy in *H. pylori* eradication.

**Patients and methods:**

In a randomized, controlled, treatment-naïve adult patients with confirmed *H. pylori* infection were assigned in a 1:1:1 ratio to one of the following 14-day open-label therapies: BQT (rabeprazole 10 mg twice daily, compound bismuth aluminate granules 2.6 g thrice daily, amoxicillin 1 g twice daily, clarithromycin 500 mg twice daily), tegoprazan-based therapies (TAD, tegoprazan 50 mg twice daily, amoxicillin 1 g thrice daily; TBQT, tegoprazan 50 mg twice daily, compound bismuth aluminate granules 2.6 g thrice daily, amoxicillin 1 g twice daily, clarithromycin 500 mg twice daily). The primary outcome was the eradication rate of *H. pylori*. Secondary outcomes included the assessment of adverse events and treatment compliance.

**Results:**

A total of 468 patients were enrolled. The eradication rates for TBQT, TAD and BQT were 86.3, 85.5 and 77.2%, respectively, by intention-to-treat analysis (*p* = 0.059), and 87.3, 87.2 and 77.7%, respectively, by per-protocol analysis (*p* = 0.029). The incidence of adverse events was comparable between the BQT and tegoprazan-based therapies (*p* > 0.05). Treatment compliance was similar across all three groups.

**Conclusion:**

Tegoprazan-based therapies achieved acceptable *H. pylori* eradication rates exceeding 85%, outperforming the BQT. Additionally, tegoprazan-amoxicillin dual therapy may serve as an alternative *H. pylori* eradication regimen in regions with high clarithromycin resistance.

**Clinical trial registration:**

http://clinicaltrials.gov, Identifier ChiCTR2300077088.

## Introduction

*Helicobacter pylori* (*H. pylori*) infection remains one of the most prevalent chronic bacterial infections globally, affecting approximately 50% of the population (1, 2). Although the incidence has been declining in developed regions, *H. pylori* continues to be a major contributor to morbidity and mortality worldwide (3). This pathogen is identified in nearly half of all adults and is strongly linked to a range of upper gastrointestinal disorders, including chronic gastritis, peptic ulcer disease, gastric mucosa-associated lymphoid tissue lymphoma, and gastric carcinoma (4). Consequently, the development of effective eradication therapies for *H. pylori* is essential for the management and mitigation of *H. pylori*-related diseases.

A multitude of therapeutic regimens have been advocated for the eradication of *H. pylori*, including triple, quadruple, and sequential therapies (5). In light of the rising incidence of antimicrobial resistance, bismuth-containing quadruple therapy (BQT), which comprising a proton pump inhibitor (PPI), clarithromycin, amoxicillin, and bismuth, has emerged as the preferred first-line treatment (6). This regimen has demonstrated high efficacy in eliminating *H. pylori*, including strains resistant to antibiotics. Nevertheless, despite the global recommendation of a 14-day BQT as both first-line and rescue therapy, failures of PPI-based treatments persist in approximately 10–30% of patients, predominantly due to antibiotic resistance (7). In China, the management of *H. pylori* is further complicated by exceptionally high infection rates, frequent adverse effects, disruptions to intestinal microbiota, and elevated recurrence rates (8). These challenges underscore the necessity for the development of modified therapeutic strategies.

Tegoprazan (TPZ) and vonoprazan (VPZ) represent novel, orally administered potassium-competitive acid blockers (P-CABs) that exhibit competitive and reversible binding to the potassium-binding site of the gastric proton pump (9). In contrast to PPIs, which necessitate activation under highly acidic conditions, P-CABs effectively inhibit the proton pump independent of acid activation (10). Furthermore, P-CABs demonstrate the capability to bind both active and inactive conformations of the proton pump (11). This dual-binding mechanism enables P-CABs to achieve maximal acid suppression more rapidly, maintains efficacy irrespective of food intake, prolongs the duration of action, and circumvents the variability introduced by CYP2C19 polymorphisms (12). Emerging evidence from recent studies indicates that P-CAB-based regimens surpass PPI-based regimens in *H. pylori* eradication rates (13). Notably, P-CAB-based regimens characterized by shorter durations and simplified drug administration protocols have achieved eradication rates that are both clinically acceptable and comparable to more complex regimens. Studies have revealed that high-dose amoxicillin-vonoprazan dual therapy attained pooled eradication rates of 85.0% in intention-to-treat (ITT) analyses and 90.0% in per-protocol (PP) analyses (14, 15). Despite these promising findings, the application of tegoprazan-based therapies for *H. pylori* eradication remains underexplored, with limited studies available to date. Future research should focus on elucidating the efficacy and optimization of tegoprazan-containing regimens to fully establish their role in *H. pylori* management.

Consequently, we initiated a prospective, single-center clinical study to assess the efficacy and safety of 14-day tegoprazan-based therapies in comparison with the 14-day BQT as first-line treatments for *H. pylori* infection. The results of this investigation indicate that tegoprazan-based therapies may provide a safer and more effective treatment alternative for patients diagnosed with *H. pylori* infection.

## Materials and methods

### Ethical approval

This study adhered to the recommendations outlined in the Consolidated Standards of Reporting Trials (CONSORT) statement for randomized controlled trials. Written informed consent was obtained from all participants prior to enrollment. The study protocol was approved by the Institutional Ethics Board of the Civil Aviation General Hospital, Beijing, China (Approval No. 2023-L-K-44). The trial was registered with the Chinese Clinical Trials Registry^1^ under registration number ChiCTR2300077088.

### Study design and participants

This study was a single-center, prospective, open-label, randomized controlled trial conducted from November 2023 to August 2024. A total of 468 eligible participants were recruited from outpatient clinics. Inclusion criteria: (1) male or female aged ≥18 years; (2) *H. pylori* infection confirmed by a positive ^13^C-urea breath test (UBT); (3) upper gastrointestinal symptoms, including epigastric pain, acid reflux, heartburn, epigastric distention, or nausea; (4) signed informed consent form. Exclusion Criteria: (1) prior receipt of standard *H. pylori* eradication therapy; (2) use of antibiotics, bismuth, or PPIs within 4 weeks prior to treatment initiation; (3) recurrent or long-term use of macrolides; (4) allergy or contraindication to study-related medications; (5) serious primary diseases; (6) hepatic or renal insufficiency; (7) alcohol abuse. Participants meeting the inclusion criteria and none of the exclusion criteria were randomized to receive the study intervention.

### Treatment regiments

Eligible patients were randomized to one of three treatment groups: (1) BQT (rabeprazole 10 mg twice daily, compound bismuth aluminate granules 2.6 g thrice daily, amoxicillin 1 g twice daily, clarithromycin 500 mg twice daily), (2) tegoprazan-amoxicillin dual therapy (TAD, tegoprazan 50 mg twice daily, amoxicillin 1 g thrice daily), (3) tegoprazan-based quadruple therapy (TBQT, tegoprazan 50 mg twice daily, compound bismuth aluminate granules 2.6 g thrice daily, amoxicillin 1 g twice daily, clarithromycin 500 mg twice daily) for 14 days. Tegoprazan, compound bismuth aluminate granules and rabeprazole were taken orally 30 min before breakfast, lunch, and dinner, while amoxicillin and clarithromycin were taken 30 min after breakfast and dinner. Administration timing was standardized to optimize treatment adherence and efficacy.

### Study outcomes

The primary endpoint was the *H. pylori* eradication rate in the three groups. Eradication was evaluated using the ^13^C-UBT conducted 4 weeks after the completion of the treatment period. The primary analysis was performed using both ITT and PP analyses. The ITT analysis included all randomized patients, with those lost to follow-up or not undergoing the ^13^C-UBT classified as treatment failures. The PP analysis included patients who achieved at least 85% drug compliance and completed the ^13^C-UBT. Drug compliance was documented through a questionnaire completed by patients.

The secondary endpoints were the adverse events (AEs) and compliance. AEs related to the study drugs were recorded daily for 14 days using a patient-completed questionnaire. When patients reported any adverse event in the questionnaire form, investigators inquired them and assessed the severity using a grading system (1–5) based on the National Cancer Institute Common Terminology Criteria for Adverse Events (NCI-CTCAE) Version 5.0. The severity of the adverse events was classified into three levels: mild (transient and well-tolerated), moderate (causing discomfort and partially interfering with daily activities), or severe (causing considerable interference with daily activities). Compliance was assessed by standardized interview at the end of treatment, as well as by pill count in the medication boxes returned at the interview. Low compliance was defined as consumption of < 85% of dispensed pills.

### Randomization and blinding

Eligible participants were randomly assigned to one of three treatment groups in a 1:1:1 ratio using block randomization with blocks of six, employing the sequentially numbered, opaque sealed envelope method (16). Allocation was managed through opaque, sealed envelopes handled by designated personnel unaffiliated with the trial. Upon enrollment, participants were sequentially provided with an envelope to determine their group assignment. To minimize selection bias and preserve the study’s integrity, outcome assessments were conducted by researchers blinded to the treatment allocations. These blinded assessors were instructed to refrain from discussing treatment specifics with participants or other investigators, ensuring that blinding was maintained throughout the duration of the study.

### Sample size calculation and statistical analysis

Based on prior studies, the eradication rates of 14-day BQT for first-line *H. pylori* treatment ranged from 70 to 85% (17, 18). As a result, this study assumed that the eradication rate was 90% in both tegoprazan-amoxicillin and tegoprazan-bismuth quadruple therapy groups. Assuming a power of 80% and an alpha of 0.025 (one-sided), and a noninferiority margin of 10%. Accounting for a 10% dropout rate, a minimum of 145 participants per group was required, resulting in a total sample size of 435 participants.

All statistical analyses were conducted utilizing the SPSS version 22.0 or R V.3.5.2 software (R Foundation for Statistical Computing, Vienna, Austria). Categorical variables are delineated by the number of patients along with their corresponding percentages, whereas continuous variables are reported as mean values accompanied by their standard deviations (SD). For the assessment of categorical data, the Chi-square test was employed, while the paired *t*-test was applied to evaluate continuous data. The non-inferiority of the two groups was assessed using the derivation of a two-sided 95% CI and one-sided *μ*-test. A *p*-value of less than 0.05 was deemed indicative of statistical significance.

## Results

### Patient enrolment and baseline characteristics

**Figure 1:**
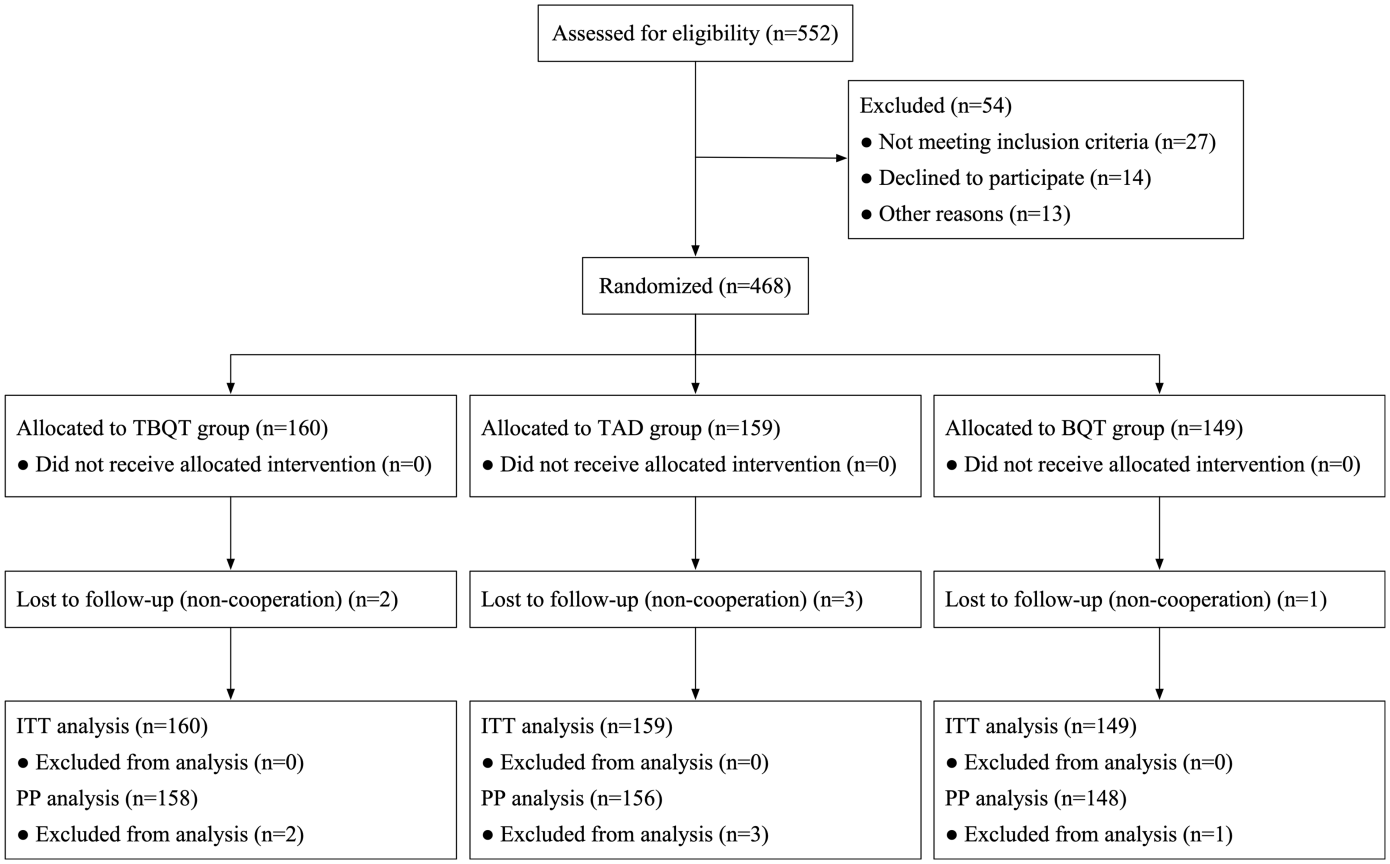
Flow chart of patient enrolment and study design. ITT, intention-to-treat; PP, per-protocol; BQT, rabeprazole 10 mg twice daily, compound bismuth aluminate granules 2.6 g thrice daily, amoxicillin 1 g twice daily, clarithromycin 500 mg twice daily; TAD, tegoprazan 50 mg twice daily, amoxicillin 1 g thrice daily; TBQT, tegoprazan 50 mg twice daily, compound bismuth aluminate granules 2.6 g thrice daily, amoxicillin 1 g twice daily, clarithromycin 500 mg twice daily.

Figure 1 delineates the patient enrollment process. Initially, 552 individuals were screened for eligibility, and finally 468 participants for the study. These participants were subsequently randomized into three distinct groups: TBQT (*n* = 160), TAD (*n* = 159), and BQT (*n* = 149). There were no significant differences in baseline characteristics across the three groups, except for a slight differences observed in the distribution of sex, with a higher proportion of males and smokers in the BQT group (Table 1). Throughout the study period, two participants from the TBQT group, three from the TAD group, and one from the BQT group lost to follow-up and consequently did not undergo the ^13^C-UBT. In the ITT analysis, these individuals were categorized as treatment failures.

**Table 1:** Baseline characteristics of study participants.

Variables	TBQT (*n* = 160)	TAD (*n* = 159)	BQT (*n* = 149)	*p*	TBQT vs. BQT; *p*	TAD vs. BQT; *p*
Age (y)	44.7 ± 13.7	44.1 ± 13.7	42.0 ± 14.1	0.198	0.085	0.191
Sex				0.157	0.087	0.059
Male	59 (36.9)	71 (44.7)	84 (56.4)			
Female	101 (63.1)	88 (55.3)	65 (43.6)			
BMI (kg/m^2^)	23.7 ± 3.5	24.0 ± 4.0	24.0 ± 3.4	0.817	0.575	0.978
Alcohol drinking	38 (23.8)	45 (28.3)	39 (26.2)	0.651	0.622	0.675
Smoking	27 (16.9)	26 (16.4)	36 (24.2)	0.152	0.112	0.088
Marital status				0.110	0.593	0.054
Married	142 (88.6)	133 (83.0)	139 (90.6)			
Unmarried	18 (11.4)	27 (17.0)	14 (9.4)			
Disease duration (m)				0.525	0.793	0.425
≤1	137 (85.6)	129 (81.1)	126 (84.6)			
>1	23 (14.4)	30 (18.9)	24 (15.4)			
Clinical condition						
Diabetes	6 (3.7)	9 (5.7)	9 (6.0)	0.615	0.349	0.887
Hypertension	16 (10.0)	20 (12.6)	11 (7.4)	0.317	0.416	0.130
Severe renal insufficiency	1 (0.6)	4 (2.5)	1 (0.7)	-	-	-
Others	19 (11.9)	22 (13.8)	10 (6.7)	0.119	0.120	0.061
DOB, median (IQR)	11.9 (5.7, 24.3)	13.1 (6.6, 25.9)	13.2 (7.4, 28.7)	0.236	0.091	0.449

Categorical variables are expressed as the number of patients, accompanied by percentages in parentheses, while continuous variables are presented as the mean ± standard deviation (SD). Disease duration is defined as the duration of patients self-reported symptoms (epigastric pain, gastric acid reflux, belching, abdominal bloating and heartburn) when outpatient visit. BMI, body mass index; IQR, interquartile range; DOB, differences over baseline; BQT, rabeprazole 10 mg twice daily, compound bismuth aluminate granules 2.6 g thrice daily, amoxicillin 1 g twice daily, clarithromycin 500 mg twice daily; TAD, tegoprazan 50 mg twice daily, amoxicillin 1 g thrice daily; TBQT, tegoprazan 50 mg twice daily, compound bismuth aluminate granules 2.6 g thrice daily, amoxicillin 1 g twice daily, clarithromycin 500 mg twice daily.

### *Helicobacter pylori* eradication rates

Eradication rates for the three treatment groups are detailed in Table 2. In the ITT analysis, the eradication rates for TBQT, TAD, and BQT were 86.3% (138/160), 85.5% (136/159), and 77.2% (115/149), respectively. Statistical comparisons revealed that TBQT significantly outperformed BQT (*p* = 0.039), while TAD approached significance compared to BQT (*p* = 0.059). Conversely, the PP analysis demonstrated eradication rates of 87.3% (138/158) for TBQT, 87.2% (136/156) for TAD, and 77.7% (115/148) for BQT. Both TBQT and TAD showed statistically significant improvements over BQT (TBQT vs. BQT, *p* = 0.026; TAD vs. BQT, *p* = 0.029; noninferiority *p* < 0.001). These findings indicate that both TBQT and TAD regimens significantly enhance the eradication rates of *H. pylori*.

**Table 2:** Eradication rates of each therapy group.

Variables	TBQT	TAD	BQT	TBQT vs. BQT		TAD vs. BQT	
				*P* for difference	*P* for noninferority	*P* for difference	*P* for noninferority
ITT	86.3 (138/160)	85.5 (136/159)	77.2 (115/149)	0.039	<0.001	0.059	<0.001
95% CI	80.9–91.6%	80.8–90.1%	70.5–83.9%				
PP	87.3 (138/158)	87.2 (136/156)	77.7 (115/148)	0.026	<0.001	0.029	<0.001
95% CI	82.4–92.1%	82.3–92.0%	70.8–84.6%				

ITT, intention-to-treat; PP, per protocol; CI, confidence interval; BQT, rabeprazole 10 mg twice daily, compound bismuth aluminate granules 2.6 g thrice daily, amoxicillin 1 g twice daily, clarithromycin 500 mg twice daily; TAD, tegoprazan 50 mg twice daily, amoxicillin 1 g thrice daily; TBQT, tegoprazan 50 mg twice daily, compound bismuth aluminate granules 2.6 g thrice daily, amoxicillin 1 g twice daily, clarithromycin 500 mg twice daily.

### Rates of AEs and compliance

Table 3 delineates the incidence rates of AEs across TBQT, TAD, and BQT groups, which were observed to be 20.6% (33/160), 15.1% (24/159), and 24.8% (37/149), respectively. Predominant AEs encompassed dizziness, abdominal pain, abdominal distention, nausea, diarrhea, and bitter taste of mouth. Importantly, no serious AEs were reported, and all other adverse events were classified as either mild or moderate in severity. Moreover, treatment adherence was notably high and did not significantly differ among the three groups, with compliance rates recorded at 97.5% (156/160) for TBQT, 98.1% (156/159) for TAD, and 97.3% (145/149) for BQT (*p* > 0.05). These findings suggest a favorable safety and compliance profile across the treatment regimens evaluated.

**Table 3:** Rates of adverse events and compliance of each therapy group.

Variables	TBQT (*n* = 160)	TAD (*n* = 159)	BQT (*n* = 149)	*p*
Adverse events	33 (20.6)	24 (15.1)	37 (24.8)	0.472
Mild	25	21	28	
Moderate	8	3	9	
Severe	0	0	0	
Adverse events				
Dizziness	4 (2.5)	2 (1.3)	4 (2.7)	0.184
Abdominal pain	5 (3.1)	2 (1.3)	6 (4.0)	0.318
Abdominal distention	5 (3.1)	6 (3.8)	8 (5.4)	0.579
Nausea	6 (3.8)	5 (3.1)	6 (4.0)	0.241
Diarrhea	3 (1.9)	4 (2.5)	5 (3.4)	0.427
Bitter taste of mouth	8 (5.0)	2 (1.3)	7 (4.7)	0.273
Others	2 (1.3)	3 (1.9)	1 (0.7)	0.541
Treatment compliance	156 (97.5)	156 (98.1)	145 (97.3)	0.709

Data are expressed as number (percentage) of patients. Treatment compliance was defined as the proportion of patients who consumed at least 85% of study drugs. AEs, adverse events; BQT, rabeprazole 10 mg twice daily, compound bismuth aluminate granules 2.6 g thrice daily, amoxicillin 1 g twice daily, clarithromycin 500 mg twice daily; TAD, tegoprazan 50 mg twice daily, amoxicillin 1 g thrice daily; TBQT, tegoprazan 50 mg twice daily, compound bismuth aluminate granules 2.6 g thrice daily, amoxicillin 1 g twice daily, clarithromycin 500 mg twice daily.

## Discussion

This study represents the assessment of the efficacy and safety profiles associated with 14-day tegoprazan-based therapeutic regimens in comparison to BQT for the eradication of *H. pylori*. In the PP analysis, both the TBQT and TAD demonstrated eradication rates of 87.3 and 87.2%, respectively. These rates were statistically superior to those observed with BQT, which achieved a 77.2% eradication rate (*p* < 0.01). Furthermore, tegoprazan-based therapies maintained high levels of patient compliance, with no significant differences noted between the regimens. Collectively, these outcomes endorse the consideration of tegoprazan-based therapies, particularly the tegoprazan-amoxicillin therapy, as viable options for the management of *H. pylori* infections.

Triple therapy, which includes a PPI alongside two antimicrobial agents, typically clarithromycin combined with amoxicillin or metronidazole, has served as the standard first-line eradication regimen for *H. pylori* infection (19). However, owing to antibiotic resistance, particularly to clarithromycin alone or in combination with metronidazole, constitutes a significant barrier to the global success of *H. pylori* eradication (20). In regions characterized by high antibiotic resistance, the efficacy of standard triple therapy has markedly declined, with eradication rates now ranging between 50 and 70% (21). The persistent failure to eradicate *H. pylori* is strongly associated with elevated gastric acidity and antibiotic resistance. Antibiotic activity is substantially enhanced when gastric acidity is neutralized; therefore, maintaining a gastric pH above 5 is recommended to optimize the effectiveness of antibiotics in eradicating *H. pylori* (22). Despite this, currently available PPIs generally do not achieve the necessary level of acid suppression consistently over a 24-h period to maintain the targeted pH level (23). P-CABs have emerged as viable alternatives to conventional PPIs. Currently, the principal P-CABs utilized in *H. pylori* eradication therapy are VPZ and TPZ (24). Compared to PPIs, P-CABs demonstrate superior and sustained gastric acid suppression, with the additional advantage of efficacy independent of cytochrome P450 2C19 (CYP2C19) polymorphism (25). Clinical evidence indicates that both VPZ- and TPZ-based regimens achieve significantly higher eradication rates than PPI-based quadruple therapy, while maintaining comparable safety profiles (26, 27). However, large-scale randomized controlled trials are warranted to conclusively establish the superior efficacy of P-CAB-based regimens relative to BQT.

The superior eradication rates observed in this study with tegoprazan dual and quadruple therapies may be ascribed to the robust gastric acid suppression of tegoprazan and its capacity to sustain elevated gastric pH levels (28). In comparative analyses (21), tegoprazan-containing quadruple therapy demonstrated eradication rates of 90.3% (modified ITT) and 90.2% (PP). In contrast, quadruple therapies based on PPIs achieved eradication rates of 84.5% (modified ITT) and 82.4% (PP). These findings indicate that tegoprazan-based quadruple therapy significantly outperforms PPI-based regimens in *H. pylori* eradication, underscoring the acid-suppressive superiority of P-CABs in therapeutic interventions against this pathogen (28). Although existing studies indicate TPZ offers faster onset and sustained acid suppression versus VPZ, its shorter mean elimination half-life (3.7–5.4 h) contrasts with VPZ’s longer terminal half-life (mean 7.7 h) (12). This pharmacokinetic profile of VPZ may contribute to its superior *H. pylori* eradication rates relative to TPZ. Given the current lack of direct comparative evidence, large-scale randomized controlled trials are warranted to assess the efficacy and safety of TPZ versus VPZ for *H. pylori* eradication. This study demonstrated a marginally higher eradication rate with tegoprazan-based quadruple therapy compared to tegoprazan-based dual therapy (ITT: 86.3% vs. 85.5%; PP: 87.3% vs. 87.2%). This observed difference may be potentially attributable to the inclusion of bismuth within the quadruple regimen. Bismuth, a gastric mucosa protector, is another crucial component of the quadruple therapy for *H. pylori* eradication. Its advantages include non-resistance and high safety in short-term applications, and its ability to improve the eradication rate of antibiotic-resistant bacteria (29, 30). Currently, BQT is recommended as the primary empirical treatment for *H. pylori* eradication by the fifth Chinese national consensus report on *H. pylori* infection management (31).

Another plausible explanation for the high eradication rates observed with tegoprazan-dual and tegoprazan-quadruple therapies pertains to the susceptibility of the *H. pylori* strains involved, which exhibited no resistance to amoxicillin. Notably, the resistance rate of *H. pylori* to amoxicillin remains significantly lower compared to other antibiotics, a factor that is often underemphasized in the development of *H. pylori* treatment regimens (32). In China, amoxicillin resistance is particularly minimal, consistently reported to be maintained at approximately 5% (33). Tegoprazan plays a crucial role in reducing the minimal inhibitory concentration (MIC) of amoxicillin by ensuring sustained acid suppression, thereby maintaining the gastric pH above 6 for the majority of the treatment period (34). This maintenance of elevated gastric pH enhances the stability of amoxicillin, allowing the antibiotic to exert its full therapeutic efficacy. The effectiveness of tegoprazan is further evidenced by its ability to enhance the sensitivity of antimicrobials, such as amoxicillin, against *H. pylori*. The pharmacodynamic properties of amoxicillin are significantly influenced by both temporal factors and pH levels (35). For instance, achieving sustained and effective blood concentrations above the MIC typically requires dosing regimens of 500 mg four times daily or 750 mg three times daily (36). However, in the present study, the tegoprazan-dual regimen, which included the administration of amoxicillin at a dosage of 1,000 mg tid, successfully prolonged maintenance facilitated the bactericidal effects of amoxicillin, enhancing its overall efficacy against *H. pylori*. Tegoprazan-based therapies as first-line treatments for *H. pylori* infection were initially reported in the early 2020s and are comprehensively summarized in Table 4.

**Table 4:** Studies of TPZ-based therapies as first-line *Helicobacter pylori* treatment.

Studies	Study design	Sample size	Regimen	Duration (days)	Eradication rates (%)	
					ITT	PP
Choi YJ et al. (19)	RCT	175	TPZ 50 mg bid, AMO 1 g bid, CLA 500 mg bid	7	62.9	69.3
Park CH et al. (43)	Retrospective	435	TPZ 50 mg bid, AMO 1 g bid, CLA 500 mg bid	14	78.6	85.5
Jung BW et al. (44)	Retrospective	620	TPZ 50 mg bid, AMO 1 g bid, CLA 500 mg bid, MET 500 mg bid	10	74.7	88.0
Kwon YH et al. (45)	RCT	79	TPZ 50 mg bid, AMO 1 g bid, CLA 500 mg bid, MET 500 mg bid	10	90.5	96.2
Jung YS et al. (46)	Retrospective	344	TPZ 50 mg bid, AMO 1 g bid, CLA 500 mg bid	14	76.7	83.4
Kim JS et al. (26)	RCT	108	TPZ 50 mg bid, TRE 500 mg qid, MET 500 mg tid, B 300 mg qid	14	80.0	90.2
Park CH et al. (47)	Retrospective	551	TPZ 50 mg bid, AMO 1 g bid, CLA 500 mg bid	14	76.4	84.5
Park CH et al. (47)	Retrospective	377	TPZ 50 mg bid, AMO 1 g bid, CLA 500 mg bid, MET 500 mg bid	10	85.9	91.1
Kong Q et al. (48)	RCT	184	TPZ 50 mg bid, AMO 750 mg qid	14	85.8	88.2
Lee JW et al. (49)	RCT	202	TPZ 50 mg bid, AMO 1 g bid 5 days, CLA 500 mg bid and MET 500 mg bid 5 days	10	87.1	87.1
Lin X et al. (50)	RCT	107	TPZ 50 mg qd, AMO 1 g tid	14	86.0	91.0
Lin X et al. (50)	RCT	107	TPZ 50 mg bid, AMO 1 g tid	14	86.0	93.8
Cho JH (51)	Retrospective	195	TPZ 50 mg bid, AMO 1 g bid, CLA 500 mg bid	14	82.9	95.8
Cho JH (51)	Retrospective	111	TPZ 50 mg bid, AMO 1 g bid, CLA 500 mg bid, B 300 mg bid	14	71.8	87.5
Cho JH et al. (34)	RCT	57	TPZ 50 mg bid, AMO 1 g bid, CLA 500 mg bid, B 300 mg bid	14	82.5	95.9
Cho JH et al. (34)	RCT	57	TPZ 50 mg bid, AMO 500 mg qid, CLA 500 mg bid, B 300 mg bid	14	87.7	98.0
Kim JY et al. (52)	RCT	193	TPZ 50 mg bid, AMO 1 g bid, CLA 500 mg bid, B 300 mg bid	7	78.8	88.9
Present study	RCT	160	TPZ 50 mg bid, AMO 1 g tid	14	85.5	87.2
Present study	RCT	159	TPZ 50 mg bid, AMO 1 g bid, CLA 500 mg bid, B 300 mg tid	14	86.3	87.3

ITT, intention-to-treat; PP, per-protocol; RCT, randomized controlled trial; AMO, amoxicillin; CLA, clarithromycin; MET, metronidazole; TRE, tetracycline; B, bismuth; TPZ, tegoprazan; qd, once daily; bid, twice daily; tid, three times daily; qid, four times daily.

The eradication failures documented in this study likely stem from the escalating prevalence of antimicrobial resistance, a factor not directly assessed herein. The surge in antibiotic resistance, alongside the heterogeneous virulence profiles of *H. pylori* strains, has profoundly undermined the effectiveness of conventional eradication protocols. In Beijing, primary resistance rates to commonly employed antibiotics, amoxicillin, clarithromycin, metronidazole, levofloxacin, and moxifloxacin, were reported at 0.7, 55.2, 68.0, 49.7, and 64.5%, respectively, with secondary resistance rates escalating to 3.2, 96.7, 90.7, 93.1, and 80.0% (37). Clarithromycin, a fundamental component of first-line eradication therapy, exhibits notably high primary resistance in Beijing, presenting a substantial obstacle (38). Clarithromycin resistance has been associated with a 40–50% reduction in eradication success rates (39). Additionally, our study revealed no statistically significant differences (*p* > 0.05) in sex distribution, with the BQT group demonstrating higher proportions of male and smoking. Our prior research demonstrated a significant association between smoking and reduced *H. pylori* eradication rates (40). However, some studies have reported no association between *H. pylori* infection and smoking. A meta-analysis found no evidence supporting an association between smoking and *H. pylori* seropositivity (41). Wu et al. (42) indicated no significant difference in smoking between *H. pylori* when analyzing combined male and female participants.

This study is subject to several limitations that warrant consideration. Firstly, the antibiotic resistance profile of *H. pylori* was not assessed, which may have influenced the evaluation of eradication rates, given that antibiotic resistance is a pivotal factor affecting treatment efficacy. Secondly, three of the treatment regimens included amoxicillin, thereby excluding individuals who are either resistant to amoxicillin or possess a penicillin allergy, which limits the applicability of these regimens to the broader patient population. Additionally, the research was conducted at a single center with a relatively small sample size, potentially restricting the generalizability of the findings. Moreover, the single-center design introduces the possibility of selection bias, which cannot be entirely ruled out and may affect the study’s overall validity.

## Conclusion

A 14-day dual therapy regimen comprising tegoprazan and amoxicillin achieved acceptable eradication rates for *H. pylori*, demonstrating efficacy comparable to tegoprazan-based quadruple therapy as a first-line eradication strategy in Beijing, China, a region characterized by high clarithromycin resistance. Notably, tegoprazan-amoxicillin dual therapy offers several advantages, including the use of a single antibiotic and reduced antibiotic consumption, thereby positioning it as a promising alternative regimen for patients with *H. pylori* infection.

## Data Availability

The original contributions presented in the study are included in the article/supplementary material, further inquiries can be directed to the corresponding author.

## Footnotes

^1^ www.chictr.org.cn
